# Pediatric Hand Surgery Training: A Spectrum of Educational Resources

**DOI:** 10.1016/j.jhsg.2024.04.004

**Published:** 2024-05-18

**Authors:** Hannah E. Korah, Amber Leis, Aaron Berger, Michael G. Galvez

**Affiliations:** ∗ University of Arizona College of Medicine, Tucson, AZ; † Department of Plastic Surgery, UC Irvine School of Medicine, Orange, CA; ‡ Division of Plastic Surgery, Pediatric Hand Program, Nicklaus Children's Health System, Miami, FL; § Pediatric Hand Surgery, Division of Pediatric Plastic and Hand Surgery, Valley Children's Healthcare, Madera, CA

**Keywords:** Fellowship, Pediatric hand surgery, Residency

## Abstract

Treatment of children with upper-extremity trauma, congenital hand differences, cerebral palsy, and brachial plexus birth injuries requires specialized training, given the spectrum of pathology and complexities of treating an individual who is still developing. Although a limited number of dedicated pediatric hand surgery fellowships are available, mastering the large breadth of the field should ideally begin early in training and may take several different pathways. The authors seek to provide a comprehensive list of resources for trainees interested in pediatric hand surgery, including training opportunities, educational tools, and networking organizations. By shining a light on these pediatric hand surgery resources, we hope to encourage future trainees to plan ahead, so that they are well-prepared for the care of children with complex upper limb reconstructive needs.

Pediatric hand surgery is a subspecialty within hand surgery that focuses on the diagnosis, treatment, and management of a wide array of hand and upper limb conditions in infants, children, adolescents, and young adults.^1^ Pediatric hand surgery encompasses both acquired and congenital hand differences. Acquired pediatric hand conditions include traumatic injuries to the hand, wrist, and upper limb; brachial plexus injuries, both birth-related and traumatic; neuromuscular disorders, and tumors. Congenital hand differences include syndactyly, polydactyly, amniotic band syndrome, thumb hypoplasia, radial longitudinal deficiency, and multiple other diagnoses.^2^ Socioeconomic and geographic factors have been shown to significantly contribute to disparate access to pediatric hand surgery across the United States.^3^^,^^4^ Pediatric hand surgical care may vary based on the region and institution, with availability frequently associated with a children’s hospital. A comprehensive approach for children may include an interdisciplinary team including the surgeon, hand therapist, social worker, physiatrist, radiologist, anesthesiologist, advanced practice providers, and nursing teams.^2^ Currently, there are minimal demographic data available on pediatric hand surgeons and where they practice. This is likely due to the historically smaller nature of the field and the varying definitions of who identifies specifically as a pediatric hand surgeon and which pathway of training they may have taken.^5^ Therefore, the path to becoming a pediatric hand surgeon can be unclear and intimidating to trainees interested in this field. This article aims to outline the various routes and resources to becoming a pediatric hand surgeon.

## Training

Training required to become a pediatric hand surgeon involves three phases, all of which must be completed to practice as a pediatric hand surgeon.

### Medical school

The first requirement in the roadmap is completing a 4-year medical school education. However, many students choose to take added years prior to residency applications, usually pursuing research experiences or an additional degree. This choice has become increasingly common as the United States Medical Licensing Examination Step 1 Board Exam transitioned to pass/fail rather than scored, making research endeavors and extracurricular activities more important aspects in assessing the qualifications of a residency applicant.^6^ Medical school could be the first exposure that a student has to the field of pediatric hand surgery, although the focus of medical school is typically on the foundations of medical care and often does not necessarily include exposure to specialties such as pediatric hand surgery.

### Residency training

Upon completing medical education, students can choose to apply to general surgery, orthopedic surgery, or integrated plastic and reconstructive surgery (PRS) residency programs on the path to becoming a pediatric hand surgeon. General surgery residency training requires a minimum of 5 years of training, with a typical focus on the gastrointestinal tract and trauma.^7^ Orthopedic surgery residency training includes 5 years of training focused on musculoskeletal pathology and restoration of function of the extremities and spine.^8^ Integrated PRS residency training is composed of a 6- to 7-year program that focuses on reconstructive and aesthetic surgeries including microsurgery, craniofacial, breast, hand, lower extremity, and microsurgery and encompasses a wide breadth of technical skill acquisition.^9^ On the other hand, independent PRS residency requires prerequisite completion of one of many surgical residency programs (general surgery, otolaryngology, neurosurgery, urology, orthopedic, or oral maxillofacial surgery) prior to completing 3 years of dedicated PRS fellowship training.^10^ Among these, various programs have residency training with or without an incorporated research track, requiring 1–2 additional years of residency training dedicated to research. The residency path that is chosen will determine the breadth of exposure to pediatric hand surgery: this usually depends upon faculty that are part of the residency program’s staff and the availability of, or affiliation with, a children’s hospital. If there is no pediatric hand surgery rotation available, then, the trainee may seek to gain this experience through their elective time at outside institutions. Residency training may provide little exposure to pediatric hand surgery, and as such, it may not be until fellowship that surgeons gain a desire to pursue this field of practice.

### Fellowship training

Following completion of residency training, candidates can then apply to a 1-year Accreditation Council for Graduate Medical Education (ACGME)–accredited Hand and Upper-Extremity Fellowship to complete their training in the path to becoming a hand surgeon.^11^ Orthopedic surgeons are uniquely eligible for an additional year to train in pediatric orthopedic fellowships, many of which can include training in pediatric hand surgery. Following hand fellowship, any graduate of an accredited program may seek to gain an additional 6-month to 1-year fellowship training specialized in pediatric hand surgery. The currently available pediatric hand surgery fellowship programs in the United States are outlined in Table 1, with information regarding geographical location, institutional affiliations, and each program’s official webpage for further details. It becomes important to consider one’s individualized training to determine what experiences are required to become a comprehensive hand surgeon with sufficient diversity of training experience to become comfortable with the reconstructive needs of the pediatric upper limb.^12^ Mentorship is an important, and often underestimated, component of this process. A good mentor can help guide the trainee through the process, ensuring that the trainee acquires meaningful training experiences that will result in an ability to comprehensively manage complex pediatric upper limb problems, including a high level of comfort in both bone and soft tissue reconstruction.

**Table 1:** Pediatric Hand Surgery Fellowship Programs∗

Program	Shriner’s Children’s Northern California	Texas Scottish Rite Dorothy & Bryant Edwards Fellowship	Cincinnati Children’s Hand Fellowship	Pediatric Hand and Upper Extremity Fellowship Program at Children’s Healthcare of Atlanta	Shriners Hospital Pediatric Hand and Upper Limb Fellowship	Texas Children’s Hospital
Location	Sacramento, California	Dallas, Texas	Cincinnati, Ohio	Atlanta, George	Philadelphia, Pennsylvania	Houston, Texas
University Affiliation	University of California, Davis	University of Texas Southwestern	University of Cincinnati	Emory University	Temple University	Baylor University
Websites	https://www.shrinerschildrens.org/post/pediatric-hand-and-upper-extremity-surgery-fellowship	https://scottishriteforchildren.org/research-and-education/education/fellowships-and-graduate-programs	https://www.cincinnatichildrens.org/education/clinical/fellowship/ortho-surgery/specialties/hand	https://www.choa.org/medical-professionals/education-and-training/fellowships-and-residencies/hand-and-upper-extremity	https://www.littlearms.org/online-application	https://www.texaschildrens.org/departments/hand-program

∗ Table 1 outlines the most up-to-date information on currently available pediatric hand surgery fellowship programs only in the United States. This table provides information on the program name, geographical location, university affiliation, and the official websites associated with each program to access further information.

## Methods

### Search strategy

A systematic search using internet search engines was conducted to obtain the information provided in this study. Key phrases searched include “pediatric” + “congenital” + “hand” + “surgery.” Google and PubMed were the predominantly used search engines. Of the resources identified, only specific and relevant findings about pediatric hand surgery were included.

### Inclusion and exclusion criteria

Resources relevant to general hand surgery were only excluded if they were not inclusive of pediatric populations. Resources were excluded if they predominantly focused on other areas of general surgery, orthopedic surgery, or PRS; these include, but are not limited to, the textbooks “Michigan Manual,” “Essentials of Plastic Surgery,” and “Netter’s Concise Orthopaedic Anatomy.” Although we acknowledge that these are valuable educational resources, we independently concluded that they were not sufficiently specific to pediatric hand surgery, and thus, they are outside of the scope for inclusion in pediatric hand surgery training. In addition, the pediatric hand surgery group, Carpal Coalition, encompassing over 30 pediatric hand surgeons, was queried to ensure additional resources were not overlooked.

## Resources

A large array of educational and mentorship resources is currently available on pediatric hand surgery. Table 2 provides a summary of a wide assortment of educational resources specifically pertinent to pediatric hand surgery, ranging from textbooks and academic journals to social media accounts, mobile applications, and more. Table 3 outlines societies and organizations dedicated to advancing knowledge and awareness of pediatric hand surgery, a tool especially important to trainees in networking to find proper mentorship within the field. Additionally, currently active support groups, awareness days, and other patient-centric resources for pediatric hand patients are outlined in Table 4. Together, these tables provide a curated selection of comprehensive resources to equip trainees and trained surgeons in advancing professional development in the field of pediatric hand surgery.

**Table 2:** Pediatric Hand Surgery Educational Resources∗

Textbooks (Currently Available to Purchase)	Radiographic Atlas of Skeletal Development of the Hand and Wrist	Surgical Approaches: Pediatric Hand Trauma	The Care of Congenital Hand Anomalies	Pediatric Hand and Upper Limb Surgery: A Practical Guide	Congenital Anomalies of the Upper Extremity: Etiology and Management	The Pediatric Upper Extremity	Pediatric Hand Surgery
Academic Journals	The Bone and Joint Journal	Journal of Hand Surgery (American volume)	Journal of Hand Surgery Global Online	Hand	Journal of Pediatric Orthopaedic Society of North America	Journal of Hand Surgery (European Volume)	
Websites or Blogs	Pediatrichandstudygroup.org	Congenitalhand.wustl.edu	Littlearms.org	Orthobullets.com	Microsurgeon.org		
Social Media and Mobile Applications	Instagram: @pediatrichand	Instagram: @thecongenitalhand	Facebook: International Microsurgery Club	Mobile Application: ASSH Hand.e			
Podcasts	The Upper Hand Podcast	Hand-P podcast	The Orthobullets Podcast	The Loupe	The Resident Review		
Global Health	Touching Hands Project	ReSurge	The American Society for Surgery of the Hand International Traveling Fellowship				
Webinars and YouTube	Little Arms	Pediatric Upper Limb & Microsurgery talks	Boston Children’s Hospital Congenital Hand Differences	Boston Children’s Hospital Pediatric Hand Exam	ASSH Pediatric Hand Symposium		

∗ *Textbooks specific to Pediatric Hand Surgery have been compiled in*Table 2*.* The *Radiographic Atlas of Skeletal Development of the Hand and Wrist* written by Sarah Idell Pyle and William Walter Greulich provides comprehensive radiographic imaging of the hand at various stages of skeletal growth. *Surgical Approaches: Pediatric Hand Trauma* by Roger Cornwall and Kevin J. Little is a guide for health care practitioners and trainees directed at describing surgical techniques used to treat hand injuries in the pediatric population. *The Care of Congenital Hand Anomalies* by Marybeth Ezaki, Michelle James, Andrea Bauer, and Lindley Wall addresses the diagnosis and management of a variety of congenital upper limb differences. *Pediatric Hand and Upper Limb Surgery: A Practical Guide* by Peter M. Waters and Donald S. Bae equips surgeons with technical knowledge for procedures commonly used to treat hand and upper-extremity differences and trauma. *Congenital Anomalies of the Upper Extremity: Etiology and Management* by Donald R. Laub, Jr. provides insight into the causes and origins of congenital hand and upper-extremity differences, while also covering the diagnosis and management of these conditions. And finally, *The Pediatric Upper Extremity* edited by Joshua M. Abzug, Scott H. Kozin, and Dan A. Zlotolow shares an orthopedic surgery focused guide on addressing upper-extremity cases in pediatric patients. *Pediatric Hand Surgery* edited by Dr Giorgio Pajardi illustrates and describes a wide range of surgical procedures in infants and children with congenital and traumatic dysfunctions with a specific focus on the impact of young age on the described pathologies. *The common academic research journals that include Pediatric Hand Surgery research are also listed in*Table 2*. The Bone and Joint Journal* is an orthopedic surgery journal that covers a range of topics from clinical research, reviews and surgical techniques. Often, the journal features articles that focus on musculoskeletal disorders, joint surgeries, sports medicine and trauma. The *Journal of Pediatric Orthopaedic Society of North America* is affiliated with the Pediatric Orthopaedic Society of North America. This journal reports an array of research in orthopedic surgery with a focus on the pediatric population, including but not limited to congenital differences, developmental disorders, musculoskeletal infections and trauma from a surgical intervention perspective. The *Journal of Hand Surgery* has the American, European and Global Online editions. The American volume is affiliated with the American Society for Surgery of the Hand (ASSH), whereas the European volume is affiliated with the Federation of European Societies for Surgery of the Hand. All three are peer-reviewed journals with a focus on their respective regions that cover a variety of hand, both pediatric and adult, research. The Hand journal is affiliated with the American Association for Hand Surgery and is an international journal that publishes on cases, pathologies, and management of the hand and upper limb. *The websites and blogs included in*Table 2 are pediatrichandstudygroup.org, congenitalhand.wustl.edu, and littlearms.org. Pediatric Hand Study Group found at pediatrichandstudygroup.org is an association of interprofessional health care providers who treat anomalies of the hand and upper limb in children. It was founded in 1995 by orthopedic trained surgeons Dr Michelle James, the nation’s first full-time pediatric hand surgeon, and Dr H Relton McCarroll.^13^ The group has a focus on research and international fellowship and is also known for the renowned annual Manske Award, honoring the late Dr Paul Mankse, that recognizes the most impactful pediatric hand and upper limb research publication. Congenital Hand and Arm Differences found at congenitalhand.wustl.edu is a blog affiliated with Washington University School of Medicine in St. Louis founded and authored by Dr Charles Goldfarb. Through his blog, he shares stories, cases, and reflections to demystify congenital and acquired differences in hand and upper extremities of pediatric populations. *Little Arms* found at littlearms.org, collated by Dr Dan Zlotolow, is a consortium of pediatric hand surgeons dedicated to educating parents and health care practitioners on the most up-to-date diagnoses, surgical approaches, and therapeutic management of the ever-evolving field of pediatric hand and upper-extremity differences. This site features a library of lectures and surgical videos hosted on YouTube. Table 2 also includes orthobullets.com and microsurgeon.org, which are not specific to pediatric hand surgery, but serve as trainee-friendly educational resources that may include pediatric pathologies. Orthobullets.com is an orthopedic surgery medical education platform that provides research articles, case presentations, educational videos, surgical technique guidelines and practice questions. Microsurgeon.org has detailed illustrations by Dr Rudy Buntic depicting various microsurgery and reconstruction focused plastic surgery techniques, as well as speaker series on a variety of pediatric microsurgery topics from the Buncke Clinic. *Social media has been widely accepted as an education tool in Hand Surgery, and thus, accounts and applications relevant to Pediatric Hand Surgery have been included in*Table 2*.* On Instagram, @pediatrichand and @thecongenitalhand, on Facebook the International Microsurgery Club, and ASSH Hand.e as a mobile application, all focus on pediatric hand diagnoses, treatment and outcomes in an on-the-go mobile access format. *Additionally, although there are currently no podcasts available specific to Pediatric Hand Surgery, five podcasts that discuss the topic in certain episodes and cover other hand, orthopedic or plastic surgery topics ideal for trainees are also shared in*Table 2*.* These include the Upper Hand Podcast by Drs Charles Goldfarb and Christopher Dy at Washington University, Hand-P affiliated with ASSH, The Orthobullets podcast affiliated with orthobullets.com, a plastic surgery podcast called *The Loupe* co-founded by Drs Morgan Martin and Greta Davis, and The Resident Review which is plastic surgery focused. *For global health experiences with Pediatric Hand Surgery as listed in*Table 2, the Touching Hands Project affiliated with ASSH offers volunteer orthopedic care at a global level with roughly seven trips annually to underserved communities abroad. This mission sends interdisciplinary teams of hand surgeons, anesthesiologists, nurses and hand therapists to underserved communities to treat acquired and congenital hand deformities in adult and pediatric patients. ReSurge has volunteers from a variety of academic institutions, including Stanford University, Johns Hopkins, and others. The foundation of ReSurge is rooted in providing sustainable reconstructive care to low-income countries by not only directly treating patients, but also, providing hands-on and virtual surgical training to health care providers in low-income countries to allow for long-term access to care and capacity building. The American Society for Surgery of the Hand International Traveling Fellowship is designed to train aspiring hand surgeons in the United States by allowing them to experience brachial plexus surgery, replantation surgery, and reconstructive microsurgery at reputable institutions abroad in China and India.^14^*Webinarsand YouTube channels that cover educational content on pediatric hand surgery are also included in*Table 2*.* The Pediatric Upper Limb & Microsurgery Talks are educational YouTube accounts for both surgeons and guardians of children in need for surgical intervention to treat upper limb differences. Organized by Drs Francisco Soldado, Scott Kozin, David McCombe, and Dan Zlotolow, Pediatric Upper Limb & Microsurgery talks provided talks ranges in a wide variety of pediatric hand surgery related issues, including nerve injury, tumors, trauma, and congenital deformities. Boston Children’s Hospital Congenital Hand Differences is a YouTube channel dedicated to educating trainees and guardians of pediatric patients who have congenital hand differences. Similarly, the Boston Children’s Hospital Pediatric Hand Exam is a three-part YouTube series that describes a standardized examination of the hand of a pediatric patient, including an overview, sensation, and motion. Finally, the ASSH has provided Pediatric Hand Symposiums in webinar format including continuing medical education course that assesses diagnosis, intervention management, and research of upper limb development differences. The course consists of an online syllabus, online tests, video resources and evaluations that are estimated to be completed in 4 hours.

**Table 3:** Societies and Organizations∗

General Societies	American Academy of Orthopaedic Surgeons American Council of Educators in Plastic Surgery American Society of Plastic Surgeons Pediatric Orthopaedic Society of North America
Hand Societies and Organizations	American Association for Hand Surgery American Society for Surgery of the Hand Pediatric Hand Study Group Paediatric Upper Limb Project Plexus Nexus
International Societies	British Society for Surgery of the Hand International Federation of Societies for Surgery of the Hand Pediatric Hand International Society of Surgeons
Conferences	European Symposium Pediatric Hand Surgery Narakas International Symposium on Brachial Plexus Surgery World Symposium on Congenital Malformations of the Hand and Upper Limb
Research	Congenital Upper Limb Difference Registry Landmark Papers

∗ Table 3 outlines societies and organizations in the umbrella of general surgery, orthopedic surgery, plastic surgery, hand surgery, and specifically pediatric hand surgery. *General societies relevant to hand surgery are listed.* The American Academy of Orthopaedic Surgeons was founded in 1933 to serve orthopedic surgeons with musculoskeletal education through an annual meeting, continuing medical education courses, online and in-person educational resources at the orthopaedic learning center, and scientific research. The American Council of Educators in Plastic Surgery is a plastic surgery oriented organization with a specific focus on leadership in education by providing training and educational resources not only to trainees, but also to the next generation of educators. American Council of Educators in Plastic Surgery comprises 92% of and 65% of ACGME-accredited plastic surgery program directors and program coordinators, respectively. Founded in 1931, the American Society of Plastic Surgeons represents 92% of all board-certified plastic surgeons in the United States and over 11,000 surgeons worldwide, making it the largest plastic surgery society in the world. It focuses on both cosmetic and reconstructive plastic surgery and caters to providers through education and advocacy. Established in 1984, the Pediatric Orthopaedic Society of North America is the first and largest pediatric-specific orthopedic society of North America with an annual internationally renowned meeting, physician and trainee education resources, and research specific to the pediatric population. *Among the Hand Surgery societies and organizations,*Table 3*outlines five major societies*, including the previously described American Association for Hand Surgery, ASSH, and Pediatric Hand Study Group. The Plexus Nexus is also mentioned, an organization dedicated to treating, educating on, and advocating for ending brachial plexus birth injuries. *International societies listed in*Table 3 include the British Society of the Hand, the International Federation of Societies for Surgery of the Hand (IFSSH), the Paediatric Upper Limb Project, and the Pediatric Hand International Society of Surgeons. British Society of the Hand is an education and research focused society for hand disorders and injuries in the United Kingdom. The IFSSH, formed in 1966, comprises hand surgeons around the world, hosts international educational courses, free educational surgical technique guide videos and now has a free of charge digital journal with recent publications from IFSSH members around the global. The Pediatric Upper Limb Project is a group of hand surgeons and therapists from all over Europe with a specific interest in children’s upper limb conditions. The Pediatric Hand International Society of Surgeons is an organization restricted to 40 members, invitation only, and restricted to hand surgeons. It has a bi-annual meeting where members congregate to share their interests, research, and improvements in the field of pediatric hand surgery. *The international conferences listed in*Table 3 are the European Symposium Pediatric Hand Surgery, Narakas International Symposium on Brachial Plexus Surgery, and the World Symposium on Congenital Malformations of the Hand and Upper Limb. Beginning 2008, the European Symposium Pediatric Hand Surgery has a triennial meeting in Europe for health care providers of the hand, including a workshop for hand therapists. The Narakas International Symposium on Brachial Plexus Surgery is a European international symposium focused on brachial plexus injuries. The World Symposium on Congenital Malformations of the Hand and Upper Limb is affiliated with IFSSH, ASSH and other societies to provide a space for hand surgeons to share their cases, research, and share expertise. Aside from previously listed academic journals from Table 2, Table 3*also provides two suggestions for research resources for Pediatric Hand Surgery*, including the Congenital Upper Limb Difference Registry and Landmark Papers on pediatric hand surgery, which are libraries of academic manuscripts that are key for understanding diagnoses of pediatric hand surgery and are typically provided during a pediatric hand fellowship. There is currently no established “reading list” for pediatric hand surgery.

**Table 4:** Patient Resources∗

Support Groups	Lucky Fin	United Brachial Plexus Network	Arthrogryposis Multiplex Congenita Support Inc	Camp Victory: The Marfan Foundation	Reaching for the Stars
Hand Prosthetics	The Helping Hand Project				
Awareness Weeks/Months	Limb Loss and Limb Difference Awareness Month (April)	Hand Therapy Week (June)	Brachial Plexus Injury Awareness Week (October)	World Cerebral Palsy Day (Oct 6)	National Cerebral Palsy Awareness Month (March) and Day (March 25)
International	Limbs 4 Kids (Australia)	Reach (UK)			

∗ Table 4 outlines the support groups, hand therapy projects, recognition celebrations, and international resources available to the pediatric patients and guardians of those affected by hand differences. *Thesupportgroups listed* includes the Lucky Fin Project, founded by Molly Stapelman, which is a non-profit support network for parents with children with congenital hand differences. This is a worldwide network that links parents to medical education on resources, limb difference awareness, and financial support for special camps, prosthetics, and other community aids for the families of and patients with limb differences. The United Brachial Plexus Network is a registered non-profit organization that strives to educate and support families of those with brachial plexus injuries, both traumatic and birth-related, worldwide as well as providing a support network for patients with these diagnoses. Arthrogryposis Multiplex Congenita Support Inc (AMCSI) is an organization dedicated to supporting those experiencing this umbrella termed diagnosis of roughly 400 types of syndromes. AMCSI especially targets new parents or soon-to-be parents in educating them on the diagnosis, as well as networking to connect to medical resources, occupational therapists, physical therapists, and more. Camp Victory: The Marfan Foundation is aimed at improving the quality of life of those with a variety of aortic and vascular diseases, including Marfan syndrome, Loeys-Dietz syndrome and Ehlers Danlos syndrome, an array of differences that can affect the upper limb. Reaching for the Stars is a non-profit organization dedicated to bettering the lives of those with and families of those with Cerebral Palsy through research, treatments, and awareness.^15^Table 4*also speaks of a group dedicated to hand prosthetics.* The Helping Hand Project uses 3-dimensional printing to provide cost-free recreational prosthetic devices to children with hand differences, while also providing a platform for these children to gain a sense of belonging and community among others like them. Additionally, *the six annual awareness celebrations shared in*Table 4 include Limb Loss and Limb Differences Awareness Month (April), Hand Therapy Week in the first week of June, the Brachial Plexus Injury Awareness week that occurs every third week of October, World Cerebral Palsy Day on October 6th, National Cerebral Palsy Awareness Month in March and Day on March 25th. *The international organizations listed in*Table 4 are Limbs 4 Kids located in Australia, and Reach located in the United Kingdom.

## Discussion

There are multiple training pathways available to become a pediatric hand surgeon in the United States. Aside from the wide spectrum of pathology that exists in—and may be unique to—children, there are several considerations that make pediatric hand surgery a complex field that requires dedicated and comprehensive training. The unique complexity of pediatric hand surgery includes, but is not limited to, open growth plates, rare presentations, and small-scaled surgery that relies on technical abilities, including microsurgery, treatment of complex bony pathology, and frequently one “best chance” to intervene with surgery to correct the deformity. The complexity of the field is also directly proportional to the long clinic consultations that are often required with the child’s family. In this patient population, a comprehensive approach including pediatric hand therapy becomes increasingly important. The diversity of training pathways to enter pediatric hand surgery may be helpful for capturing key components including soft tissue handling and bony fixation; however, there may be poor overlap with the diversity of topics covered in training.^16^ Given this complexity, the goal should be for the trainee to start early and be flexible in their route to the specialty to ensure that they are knowledgeable and comfortable in treating the spectrum of acquired and congenital hand differences in children.

Each subcategory within pediatric hand surgery could have its own dedicated pathway of study. Pediatric hand trauma has entire textbooks dedicated just to this field. Congenital hand surgery is a field within itself, given the array of differences that can affect the upper limb. Cerebral palsy has the complexities of requiring comprehensive care including physiatry, pediatric orthopedic care, and hand therapy evaluation potentially requiring video assessment and is managed with an array of surgical treatments ranging from tendon transfers to selective neurectomies. Finally, brachial plexus injuries can include those that are birth-related or those secondary to a traumatic injury. Multiple pediatric hand surgery fellowships provide training in brachial plexus injuries; however, there are also dedicated peripheral nerve fellowships that are beyond the scope of this article. Currently, there is no central location to determine the peripheral nerve fellowships available, which also appear to be increasing in number.

Given the complexity of the pathology and the relative rarity of many of the pathologic processes treated in pediatric hand surgery, additional training may be necessary to broaden the pediatric hand surgeon’s knowledge base, as some experiences may not have been available during residency training, a 1-year hand surgery fellowship (where exposure to pediatric hand surgery training is variable but can sometimes be tailored to pediatric-specific problems), or even during a 6-month pediatric hand surgery fellowship. Currently, there are no established pediatric hand surgery educational guidelines or milestones to capture all diagnoses of the pediatric upper limb. This study aims to bring more transparency to the process and help informally bridge gaps by systematically describing the resources across multiple specialties to educate the current and next generation of pediatric hand surgeons. Creating this awareness will allow the next generation to be able to anticipate and plan for training opportunities.

In some pediatric subspecialties, it is believed that there may be an oversaturation of specialists trained.^17^ However, for pediatric hand surgery, as of early 2024, there were over 10 open positions in the United States (Supplementary Fig. S1, available online on the Journal’s website at https://www.jhsgo.org). In addition, there are new treatment centers that are being developed in areas of the United States that have not had access to pediatric hand surgery previously. Because diagnoses such as cerebral palsy have known barriers to care, it becomes important to have local comprehensive care available. As part of an overall goal of reducing health care disparity, the number of pediatric hand surgeons will need to increase. Unfortunately, not every children’s hospital in the United States includes a pediatric hand surgeon on staff, and therefore, it is important to advocate for adequate coverage for all children that may require this complex care locally, especially given the need for long-term care as the child grows. Transparency of the training pathways for pediatric hand surgery has not been well defined. This article provides a uniform collection of resources where additional training can be obtained including the available fellowships. As there are only five pediatric hand fellowships currently offered in the United States and clearly demonstrated clinical need, the demand for this training may be greater than the pediatric hand fellowship pathway can support. Additionally, the financial limitations presented by taking on additional formalized training may deter some trainees. Therefore, strategies for integrating hand surgery early including the Joint Plastic Surgery Hand Surgery Program, an accelerated track for both fields within 6 years, and establishing a Hand Surgery Residency in the United States, may allow for earlier integration of pediatric hand surgery topics.^18^^,^^19^

By providing educational resources, societies, and conferences in one location for those interested in pediatric hand surgery, this study may help those interested in pursuing training in this subspecialty. Additional research is needed to determine the scope of practice for each pediatric hand surgery fellowship available for the interested trainee. In addition, the available international pediatric hand surgery fellowships should also be delineated for the interested trainee including the American Society for Surgery of the Hand International Traveling Fellowship. In the past, exclusive physical libraries existed for the interested trainee; however, these were limited to specific institutions. In our current state of available information online, there is now a plethora of information that should be gathered and curated. Providing a continuously updated source of pediatric hand surgery knowledge will become important in the pathway forward to define this subspecialty. As this is the first instance in which all these resources have been collated, we acknowledge that—given the vast array of resource locations—some resources may have not been included or may no longer be readily available.

The distinction of pediatric hand surgery as a subspecialty of hand surgery becomes especially important because a pediatric patient in the United States cannot simply present to a hand surgeon and expect treatment, especially with restrictions of pediatric anesthesia availability and insurance barriers. Similar to creating transparency for training in pediatric hand surgery, it also becomes important for families to be able to find a pediatric hand surgeon with appropriate experience and training locally. Although there are several hand surgery societies currently in existence, there is no umbrella organization that provides specific training information for pediatric hand surgery nor a patient-focused resource for identification of a pediatric hand surgeon nearest to the family. Furthermore, given the sheer quantity of knowledge required for the treatment of patients with pediatric hand surgery needs, in the future, this field may further differentiate into a standalone specialty in the same way that pediatric surgery became separate from general surgery. Finally, given the wide array of journal locations for pediatric hand surgery research literature, there is easily sufficient volume for a dedicated pediatric hand surgery journal.

We hope that this comprehensive resource that has been collated, in combination with strong mentorship and sponsorship, will help future pediatric hand surgeons so that they are well-prepared for the care of children with complex upper limb reconstructive problems.

## Conflicts of Interest

No benefits in any form have been received or will be received related directly to this article.
