# Supplementary material for: Pediatric Hand Surgery Training: A Spectrum of Educational Resources
Source: J Hand Surg Glob Online. 2024 May 18;6(4):471–6. doi: 10.1016/j.jhsg.2024.04.004 (PMC11331224; doi:10.1016/j.jhsg.2024.04.004)
Supplement: Supplementary Fig. S1 [file mmc1.docx]

| **Program** | Boston Children's Hospital | Children's Hospital of Philadelphia | Phoenix Children's | Louisiana State University | Children's Mercy - Kansas City | Riley Children's - Indiana University Health | University of Utah | UC San Diego | Texas Children's Hospital | Joe DiMagggio’s Children’s Hospital |
| --- | --- | --- | --- | --- | --- | --- | --- | --- | --- | --- |
| **Department** | Ortho | Surg or Ortho | PRS | PRS | Ortho | Ortho | PRS | Ortho | Ortho | Ortho |
| **Websites or Contact information** | <https://acrobat.adobe.com/id/urn:aaid:sc:US:88c4782b-3395-4b04-902c-f540647220a4> | Contact Dr. Apurva Shah shaha6@chop.edu with a letter of interest and CV. <https://jobs.assh.org/job/pediatric-plastichand-surgeon/72486340/> | <https://acaplasticsurgeons.org/jobs/?jobID=4109> | <https://lsuhsc.peopleadmin.com/postings/15619> | <https://g.co/kgs/837oxBX> | [https://shorturl.at/blnXY](https://shorturl.at/ehknP) | <https://shorturl.at/yCM47> | <https://posna.org/resources/job-board/find-a-job/position-details?itemid=458> | Contact Dr. Chris Pederson wcpeders@texaschildrens.org with a letter of interest and CV. | Contact Dr. Stephen Plachta at Splachta@mhs.net with a letter of interest and CV. |
| **Additional Information** |  | Microsurgical expertise required | Senior level position (section chief) | Adult hand trauma with the potential for pediatric hand surgery |  |  |  |  |  |  |

**Supplemental Figure 1. Early 2024 Open Positions in Pediatric Hand Surgery**
